# Salvage Treatment for Progressive Brain Metastases in Breast Cancer

**DOI:** 10.3390/cancers14041096

**Published:** 2022-02-21

**Authors:** Mateusz Jacek Spałek, Tomasz Mandat

**Affiliations:** 1 Department of Soft Tissue/Bone Sarcoma and Melanoma, Maria Sklodowska-Curie National Research Institute of Oncology, ul. W.K. Roentgena 5, 02-781 Warsaw, Poland; 2 Department of Neurosurgery, Maria Sklodowska-Curie National Research Institute of Oncology, ul. W.K. Roentgena 5, 02-781 Warsaw, Poland; tomasz.mandat@pib-nio.pl

**Keywords:** breast cancer, brain metastases, intracranial progression, radiosurgery, stereotactic radiotherapy, whole-brain radiotherapy, reirradiation

## Abstract

**Simple Summary:**

Thirty percent of patients with human epidermal growth factor receptor 2-positive breast cancer and triple-negative breast cancer, and 15% of patients with the remaining subtypes of breast cancer will develop brain metastases. Available treatment methods include surgery and radiotherapy. However, some individuals will experience intracranial progression despite prior local treatment. This situation remains a challenge. In the case of progressing lesions amenable to local therapy, the choice of a treatment method must consider performance status, cancer burden, possible toxicity, and previously applied therapy. Stereotactic radiosurgery or fractionated radiotherapy rather than whole-brain radiotherapy should be used only if feasible. If local therapy is unfeasible, selected patients, especially those with human epidermal growth factor receptor 2-positive breast cancer, may benefit from systemic therapy.

**Abstract:**

Survival of patients with breast cancer has increased in recent years due to the improvement of systemic treatment options. Nevertheless, the occurrence of brain metastases is associated with a poor prognosis. Moreover, most drugs do not penetrate the central nervous system because of the blood–brain barrier. Thus, confirmed intracranial progression after local therapy is especially challenging. The available methods of salvage treatment include surgery, stereotactic radiosurgery (SRS), fractionated stereotactic radiotherapy (FSRT), whole-brain radiotherapy, and systemic therapies. This narrative review discusses possible strategies of salvage treatment for progressive brain metastases in breast cancer. It covers possibilities of repeated local treatment using the same method as applied previously, other methods of local therapy, and options of salvage systemic treatment. Repeated local therapy may provide a significant benefit in intracranial progression-free survival and overall survival. However, it could lead to significant toxicity. Thus, the choice of optimal methods should be carefully discussed within the multidisciplinary tumor board.

## 1. Introduction

Breast cancer brain metastases (BCBM) represent the second most frequent secondary malignancy in the brain [1]. The introduction of modern systemic therapies has helped to prolong survival of patients with breast cancer. Thus, we observe the increasing incidence of BCBM that occur mostly in patients with human epidermal growth factor receptor 2 (HER2)-positive breast cancer and triple-negative breast cancer [2,3,4,5]. In a recent meta-analysis, it was found that the pooled cumulative incidence of BCBM was around 30% for the HER2-positive subgroup and the triple-negative subgroup, and 15% among patients with hormonal receptors positive HER2-negative breast cancer [6]. The diagnosis of BCBM is related to poor prognosis, neurological deficits, and impaired quality of life. Deterioration of the performance status may lead to discontinuation of thus far effective systemic treatment and close other treatment options, including clinical trials.

In the past, whole-brain radiotherapy (WBRT) was a gold standard of treatment for BCBM, but its efficacy is disputable [7]. The introduction of advanced surgical techniques and stereotactic radiotherapy, namely, single-fraction stereotactic radiosurgery (SRS) and fractionated stereotactic radiotherapy (FSRT), opened the possibilities of focused treatment without affecting the healthy brain, resulting in better local efficacy and a significant reduction of neurocognitive impairment associated with WBRT [8,9,10]. Contemporary radiotherapy techniques allow for treating multiple brain metastases at the same time without a decrease in efficacy [11]. Furthermore, selected patients with BCBM may survive several years using available modern treatment methods [12]. However, intracranial progression of previously treated BCBM remains a challenge due to lack of guidelines and risk of treatment-related toxicity. The most challenging situations comprise local progression after surgery or SRS/FSRT and disseminated intracranial progression after WBRT.

In this narrative review, we aimed to summarize data on treatment methods and research directions of salvage treatment for progressive BCBM. We hope that it will be useful in the clinical management of these patients and will help to identify the optimal treatment methods. All analyzed papers regarding methods of salvage treatment are summarized in Table S1.

## 2. Definitions

Progressive BCBM is defined as metastatic brain tumors from breast cancer that has been treated with any form of local therapy and recurred or progressed after it. A single patient may experience a progression of a solitary lesion or multiple metastases.

Local therapies for BCBM include neurosurgery, SRS, FSRT, and WBRT [13]. SRS is delivered in a single fraction of a dose on the basis of the maximum diameter of the tumor [14]. FSRT could be given in three, five, or more fractions and is usually applied in the case of larger lesions. Interestingly, the efficacy of both SRS and FSRT seems to be similar [15]. The summary of commonly prescribed doses is presented in Table 1.

WBRT is usually delivered in 5 (5 × 4 Gy) or 10 (10 × 3 Gy) fractions. Longer regimens were found to be unfeasible due to lack of benefit in efficacy and increased frequency of adverse events [16].

## 3. Diagnosis of Intracranial Progression

The response of BM may be assessed using two systems: Response Evaluation Criteria in Solid Tumors (RECIST) and Response Assessment in Neuro-Oncology (RANO) criteria [17,18]. The advantage of RANO criteria is the inclusion of parameters associated with neurological status, the use of steroids, and the occurrence of pseudoprogression after SRS/FSRT or during immunotherapy. For example, the deterioration of neurological status is an independent criterion of intracranial progression, regardless of lack of increase in the target lesions diameters. RANO criteria are presented in Table 2.

According to the RANO criteria, the golden standard of monitoring of BM is gadolinium-enhanced T1 and T2 magnetic resonance imaging (MRI). Computed tomography should be only used exceptionally, for example, in the case of contraindications to MRI. Thin slice thickness is recommended, preferably less than 1.5 mm. The lesion should have at least 1 cm in the longest diameter. That excludes lesions with unclear borders, meningeal infiltration, bone metastases, and purely cystic lesions. The maximum number of lesions should not exceed five. The brain is treated as a separate compartment and excluded from the extracranial assessment.

Special attention must be paid to the lesions that were irradiated with SRS or FSRT. Post-radiation necrosis (PRN) could mimic the progression of the BCBM [19,20]. This situation is relatively common; thus, the significance of the problem in a cohort of breast cancer patients with better prognosis and access to other treatment options is high due to the possibility of salvage treatment [14,21,22]. Several diagnostic modalities may help to differentiate between PRN and tumor progression. That includes MRI spectroscopy, positron emission tomography, single-photon emission computed tomography, and high-resolution MRI with the assessment of perfusion and diffusion [23,24,25,26,27,28,29]. However, the only 100% reliable method of confirmation of progressive BCBM after SRS/FSRT is a pathological examination of the resected tumor. Nevertheless, its use may be limited by anatomical localization of the suspected lesion and postsurgical morbidity.

## 4. Available Options of Local Salvage Treatment in Various Clinical Situations

### 4.1. Local Progression after WBRT

In the case of limited local progression after WBRT, available treatment options include both salvage surgery and SRS/FSRT. Unfortunately, available literature on this topic consists only of retrospective data rather than clinical trials or prospective cohorts.

The research group from Heidelberg (Germany) described the results of SRS for BCBM, including 39 patients who received salvage SRS after previous WBRT [30]. Median overall survival from the time of SRS reached an impressive 19 months. Younger age (<40 years) was the only factor associated with better outcomes.

Another group from the University of Texas M.D. Anderson Cancer Center reported outcomes from a similar cohort of patients who underwent WBRT for BCBM and salvage SRS due to the intracranial progression [31]. The median overall survival was 14 months. The one and two year overall survival of the 15 analyzed patients reached 55% and 23%, respectively. One and two year local control rates in this group were 76% and 46%, respectively. Higher Karnofsky performance score (≥90), higher Score Index for Radiosurgery (≥6), estrogen receptor positivity, and post-menopausal status were found to be associated with better survival.

Kased et al. published results of SRS for recurrent BCBM after previous WBRT (*n* = 81), surgery with WBRT (*n* = 18), and surgery alone (*n* = 4) [32]. The median overall survival was 11.7 months. Younger age (<50 years), better performance status (Karnofsky performance score ≥70), extracranial control, estrogen receptor positivity, and HER2 positivity were associated with longer survival. Importantly, the number of irradiated lesions did not affect survival time.

In another cohort analysis, the authors retrospectively assessed data of 79 patients with BCBM who underwent salvage SRS after previous therapy [33]. The group included 63 patients after WBRT, 13 patients after surgery and WBRT, 1 patient after total meningeal irradiation, 1 patient after surgery, and 1 patient after sole systemic therapy. The median intracranial progression-free survival after SRS was 5.7 months, whereas median overall survival reached 9.8 months. HER2 positivity and controlled extracranial disease were prognostic factors for survival.

Huang et al. assessed a cohort of 56 patients with BCBM who were reirradiated due to the intracranial progression [34]. In the first group, 39 patients underwent WBRT as an initial treatment and salvage SRS. The second group comprised 17 patients who initially received SRS and underwent salvage WBRT after intracranial progression. Patients who received salvage SRS had 6.5 months of median intracranial progression-free survival and 11.4 months of median overall survival. Results in the second group were similar, namely, 8.5 months of median intracranial progression-free survival and 10.8 months of median overall survival. The most important prognostic factor in both groups was performance status.

In another retrospective analysis, the authors assessed outcomes of 40 patients with BCBM who experienced intracranial progression after WBRT and received secondary radiotherapy [35]. A total of 12 patients received salvage SRS, 9 patients underwent FSRT, and 19 patients were irradiated with repeated WBRT. Median survival after salvage radiotherapy was low, but still significantly better in HER2-positive patients compared with HER2-negative patients (6 months vs. 2 months; *p* = 0.015).

### 4.2. Local Progression after Local Treatment

Intracranial local progression after surgery without postoperative radiotherapy may be limited to the surgical cavity or surroundings of the tumor bed. In both situations, salvage SRS and FSRT of progressive BCBM after surgical resection of the tumor should be used as a preferred method in patients with good performance status, only if feasible. Salvage WBRT should be reserved for patients with disseminated progression.

Intracranial local progression after SRS/FSRT may occur outside or within a previously irradiated volume. In the first situation, both surgery and repeated SRS/FSRT are considerable options [36]. The most challenging situations comprise local progression within the previously locally treated volume. Salvage surgery remains the mainstay method in the case of limited progression of BCBM after SRS or FSRT when progression occurs within previously irradiated lesions or is symptomatic. In the literature, there are no retrospective or prospective studies focused specifically on patients with resected progressive BCBM. Nevertheless, several studies investigating outcomes of surgical resection of progressive brain metastases from various cancers also covered patients with BCBM.

Kano et al. presented outcomes of 58 patients who underwent metastasectomy after SRS, including nine patients with breast cancer [37]. The survival rates in the entire cohort after surgery were 65, 30, and 16% at 6, 12, and 24 months, respectively. One patient with melanoma died due to intracranial hemorrhage, whereas four developed new neurological deficits.

Another study focused on results of surgical resection of suspected progression brain metastases after Gamma Knife SRS [38]. The group consisted of 32 patients from a cohort of 245 patients with brain metastases after Gamma Knife SRS. Among them, four patients had the diagnosis of BCBM. The 6, 12, and 24 month survival rates from the date of SRS of the whole cohort of patients who underwent Gamma Knife SRS were 70, 46, and 24%, respectively. Interestingly, survival of the patients who previously underwent surgery (97%, 78, and 47%, respectively) was better than the survival of the patients without surgery (65%, 40%, and 19%, respectively; *p* < 0.0001). Moreover, in four cases, postoperative specimens showed only PRN. The complication rate after surgery was relatively high. The neurological condition of four patients deteriorated, two patients developed systemic toxicity (deep venous thrombosis), and one died from malicious brain edema.

Nine patients with BCBM were analyzed within another cohort of patients who underwent resection of progressive brain metastases after SRS [39]. The authors used a risk-adapted approach. They divided lesions into those localized in non-eloquent and eloquent brain regions. Those localized in the non-eloquent regions were resected radically by using a fence-post method. Lesions localized in the eloquent areas were removed using the piecemeal technique without margins, followed by postoperative SRT. No significant differences in local recurrence rate between the fore mentioned subgroups were found (34.6% in minimum resection ± SRS group vs. 14.3% in wide resection group, *p* = 0.07). The median overall survival after salvage surgery reached 20.2 months. What is particularly important is that primary breast cancer was found to be a good prognostic factor for overall survival (breast vs. lung: hazard ratio 0.17; breast vs. others: hazard ratio 0.08). The authors did not report any significant toxicity after surgery. This study also suggested that postoperative radiotherapy after resection of progressive previously irradiated BCBM may be justified. Unfortunately, no prospective trials on this topic exist.

**Figure 1:**
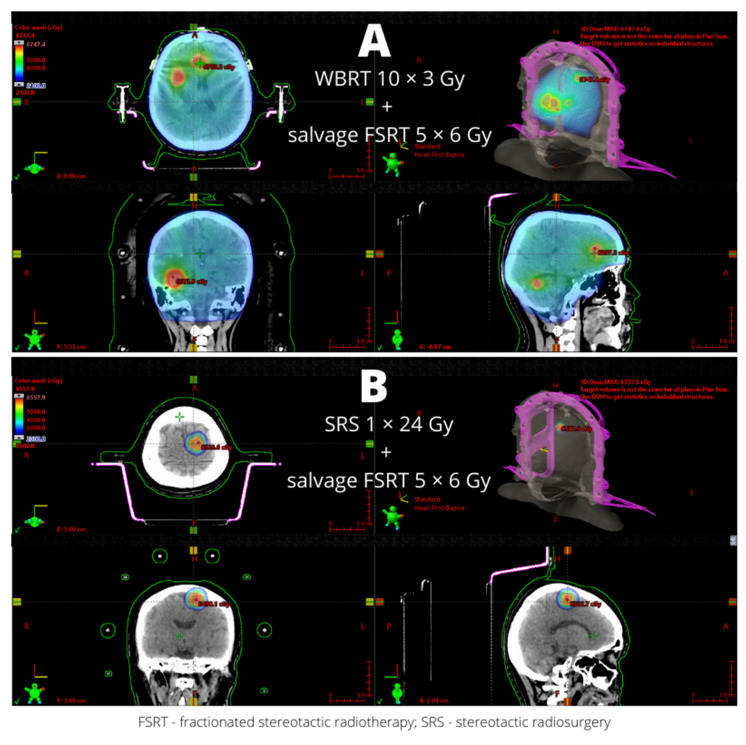
Reirradiation due to limited intracranial progression. (**A**) Salvage fractionated stereotactic radiotherapy (5 × 6 Gy) for five progressive brain metastases after whole-brain radiotherapy (10 × 3 Gy). (**B**) Repeated fractionated stereotactic radiotherapy (5 × 6 Gy) for one progressive brain lesion after prior stereotactic radiosurgery (1 × 24 Gy) in a patient who refused salvage surgery.

Repeated SRS is associated with a higher risk of PRN. It is highly advisable to make an image registration and assess a composite dose (see Figure 1).

In a study published by McKay et al., 32 patients, including 9 with BCBM, received re-SRS to 46 progressive lesions [40]. After one year follow-up, survival was 70%. The authors identified nine events of local progression that translated into 79% of one year local control for each lesion and 95.6 months of median time to local failure after re-SRS. Nevertheless, only 44% of local progression occurred within the irradiated volume. The toxicity of re-SRS was significant. In 30% of the patients, PRN was identified. In 24% of patients, PRN was symptomatic. That included dyscoordination, motor weakness, vision impairment, aphasia, and hemorrhage. Freedom from PRN at 1 year was 71%. The 10%, 20%, and 50% probabilities of PRN were associated with irradiated volumes receiving 40 Gy or higher at 0.28 cm^3^, 0.76 cm^3^, and 1.6 cm^3^, respectively.

The authors of another study on re-SRS and SRT evaluated 28 patients with 32 brain metastases who received such a treatment [41]. The cohort included five patients with BCBM. The median overall survival was 22 months. The local control rate after re-SRS was 84.4%. The one and two year local control rates were 88.3% and 80.3%, respectively. PRN was found in 18.8% of lesions. Higher prescribed isodose line (*p* = 0.033) and higher gross tumor volume (*p* = 0.015) were predictive factors for PRN in a univariate analysis.

Interesting results were provided by the authors of systematic review and meta-analysis on efficacy and safety of re-SRS or re-FSRT for brain metastases [42]. After a literature search according to PRISMA guidelines, 13 studies with 464 patients and 549 brain metastases were included. One year local control was from 46.5% to 88.3%. Pathological diagnosis of melanoma, no prior WBRT, larger tumor volume, lower given dose, inadequate response after primary SRS, poorer performance status, and uncontrolled extracranial disease were predictive for worse local control. The authors reported a crude median value of PRN as 14.3% (from 2% to 36%). Moreover, three studies included in the review described significant risk factors for PRN. This included larger irradiated tumor volume, higher given dose, the large overlap between previously and currently irradiated volumes at doses of 18 and 12 Gy, and a higher covering isodose.

### 4.3. Disseminated Intracranial Progression

Disseminated intracranial progression may occur after local treatment or WBRT. In the first situation, available salvage treatment options include WBRT and, in selected situations, local treatment. WBRT remains the method of choice in the case of numerous progressive BCBM, especially for patients with HER-2-positive and hormone receptor-positive breast cancer. However, contemporary radiotherapy techniques allow for simultaneous or sequential focal treatment of multiple progressive BCBM without loss in local efficacy [11,43,44]. Thus, even a progression of more than four BCBM is not a default indication for WBRT or best supportive care only. The choice of treatment method should rather be based on patient performance status, availability of systemic treatment options, radiotherapy equipment, the total volume of progressing tumors, and dosimetric parameters [45,46]. FSRT may be preferred over SRT if there is a significant risk of PRN [15].

In multiple BCBM, surgery can be combined with SRS/FSRT or WBRT, especially if there is a dominant lesion that causes significant edema, bleeding, or neurological symptoms [47,48]. After resection of the dominant metastatic tumor, the remaining lesions could be irradiated with or without a postsurgical bed using stereotactic methods or WBRT. However, data regarding the efficacy and tolerability of such an approach is scarce.

In the case of progression of numerous BCBM after prior WBRT, the available options of salvage treatment include repeated WBRT and systemic therapy with intracranial activity. Repeated WBRT could be offered to carefully selected patients with disseminated BCBM. The most commonly used regimens are usually more conservative than in primary WBRT, i.e., 20 Gy in 10 fractions. The tolerability of such an approach seems to be acceptable; however, the survival of patients after re-WBRT is usually poor, and data regarding late toxicity do not exist. Guo et al. reported outcomes of 41 patients treated with secondary WBRT [49]. The cohort included eight patients with BCBM. The median overall survival following re-WBRT was 3.3 months.

### 4.4. Leptomeningeal Disease

Development of leptomeningeal disease is relatively rare but is a serious event associated with poor prognosis in patients with breast cancer [50]. It is defined as the infiltration of the leptomeninges by cancer cells and their presence in the cerebrospinal fluid. It may be confirmed in MRI or cerebrospinal fluid analysis. There are no studies that directly focus on salvage treatment of secondary leptomeningeal disease after prior treatment for BCMC.

In the case of primary leptomeningeal disease, the method of choice is WBRT. However, the real benefit of such a treatment is disputable, and the prognosis remains very poor. Twenty-two patients with breast cancer and leptomeningeal metastases who underwent WBRT presented poor median overall survival [51]. Patients in good performance status and absence of solitary BCBM gained the most from WBRT. Similar poor results regarding survival after WBRT for leptomeningeal BCBM were reported by Gani et al. [52].

Leptomeningeal disease most commonly presents diffuse character. Thus, SRS and FSRT are not the methods of choice. However, in a small retrospective study, the authors analyzed a cohort of 16 patients, including 5 patients with BCBM who underwent SRT for focal leptomeningeal metastases [53]. It resulted in 10 months of median overall survival in the entire group. Six patients received salvage WBRT due to the intracranial progression. Thus, SRT and FSRT may be applied to carefully selected patients with limited localized leptomeningeal metastases who are in good performance status and are potential candidates for further systemic therapy.

### 4.5. Subtype-Tailored Treatment

In the case of multiple but countable BCBM, the choice between WBRT and SRS/FSRT remains unsolved. The use of local treatment without WBRT is associated with the higher risk of intracranial failure outside the irradiated volume. On the other hand, WBRT is related to the higher risk of neurocognitive disfunction [9]. Moreover, there is no difference in overall survival between patients who received WBRT and those who underwent SRS, regardless the higher risk of intracranial failure. SRS may be safely used, even in the case of multiple brain metastases [54,55]. Thus, repeated SRS/FSRT seems to be also a valid treatment strategy.

Breast cancer subtype may be a prognostic marker for survival following treatment for BCBM. Patients with HER-positive and triple-negative breast cancer have the poorest survival after brain radiotherapy [56]. However, in HER-2 positive BCBM, survival increases after addition of HER-2-targeted therapy to irradiation [5]. Unfortunately, there are no data on the rate of intracranial failure following SRS for each breast cancer subtype. A valuable tool to assess expected survival of patients with BCBM is the most recent version of graded prognostic assessment (GPA) [57]. It covers not only the performance status but also breast cancer subtype, age, number of BCBM, and the presence of extracranial metastases. All these factors should be considered when choosing a salvage treatment.

## 5. Salvage Systemic Therapy

The efficacy of systemic agents in the brain is limited by the blood–brain barrier (BBB) [58]. However, selected systemic therapies showed activity against BCBM [59]. If the intracranial progression occurs during a systemic treatment and there are no options of local therapy, it is reasonable to switch systemic therapy lines. Nevertheless, there are no prospective clinical trials with systemic treatment focused on patients with BCBM that progressed after local therapy. Limited data exist in the form of retrospective studies and subgroup analyses from prospective trials on patients with brain metastases.

### 5.1. All Breast Cancer Subtypes

Although many commonly used systemic agents in breast cancer do not cross the BBB, some of them present intracranial activity due to impaired vessel permeability caused by brain metastases. In a prospective nonrandomized study published by Boogerd et al., 22 patients with BCBM received either cyclophosphamide, methotrexate, and 5-fluorouracil (20 patients) or cyclophosphamide, doxorubicin, and 5-fluorouracil (two patients) chemo regimens [60]. The cohort included seven patients who had been previously locally treated for BCBM. The authors observed a significant clinical benefit in 5/7 abovementioned patients and signs of radiological response in 4/7 patients. The achieved response lasted more than 20 weeks in four of them.

Temozolomide is an alkylating agent that significantly penetrates the BBB. It is widely used in the treatment of primary and secondary brain gliomas. However, it has been also investigated in the treatment of brain metastases [61]. Results of the Hellenic Cooperative Oncology Group phase II clinical trial suggested relatively high intracranial activity of temozolomide in patients with brain metastases from solid tumors [62]. Among them, almost 50% had a diagnosis of breast cancer. The primary endpoint of this study was the response to investigated chemo regimen both in intracranial and extracranial disease. The previously treated patients received temozolomide 150 mg/m^2^, whereas chemotherapy-naïve patients were treated with temozolomide 200 mg/m^2^ for 5 days. Temozolomide was given concurrently with cisplatin 75 mg/m^2^. The cycles were repeated every 28 days. Among 15 patients with BCBM, partial response was observed in 6 of them. In the manuscript, the number of patients with BCBM who experienced stable disease or progressive disease was not stated. The treatment was well-tolerated; however, one toxic death of a breast cancer patient with hepatic and brain metastases was reported. Temozolomide was also investigated in combination with capecitabine in a prospective phase I clinical trial dedicated to patients with BCBM [63]. The primary endpoint of the study was the assessment of the maximum tolerated dose of the combination of temozolomide and capecitabine in patients with BCBM. The patients were divided into four cohorts who received investigated medications at different dosing levels on days 1–5 and days 8–12, with cycles repeated every 21 days. A total of 24 patients were enrolled, including 10 patients with progressive BCBM. The authors reported one complete and three partial responses. Half of the patients experienced stable disease. Importantly, partial responses were observed in two patients who previously underwent WBRT. The median duration of the response was eight weeks. Toxicity was typical to the investigated regimen and resulted in dose reduction among two cases. In another phase II clinical trial on patients with progressive brain metastases the from various origin, temozolomide (150 mg/m^2^ for patients who received any prior chemotherapy and 200 mg/m^2^ for chemotherapy-naïve patients) was given as a single agent. Among 41 patients, 10 had BCBM. Among four patients with BCBM, the disease was stabilized; in the other three, the progression of the disease was observed; and the remaining three patients were not eligible for analysis. The treatment was well tolerated. In summary, temozolomide seems to be an interesting option for patients with progressive BCBC. However, it is currently almost exclusively used in the treatment of malignant glioma.

Another agent is bevacizumab, a recombinant humanized monoclonal antibody against vascular endothelial growth factor. It inhibits angiogenesis, decreases the rate of contrast-enhancing lesions, reduces brain edema, and allows for the reduction in or withdrawing of steroids [64]. In a retrospective study performed at the University of Heidelberg (Germany) and the Medical University of Vienna (Austria), the authors assessed the efficacy of bevacizumab, a monoclonal antibody against vascular endothelial growth factor, as salvage therapy in patients with symptomatic, recurrent brain metastases who were not candidates for any form of local therapy [65]. The study group consisted of 22 patients, including 9 patients with BCBM. All of them were previously irradiated (WBRT or SRS), and two of them underwent brain metastasectomy. The median length of bevacizumab therapy was five months. The most frequent reason for treatment withdrawal was the progression of the disease. Neurological improvement was observed in most treated patients (64%), whereas stabilization was found in 27%. Thus, the clinical benefit occurred in 91% of patients who received bevacizumab.

### 5.2. Triple-Negative Breast Cancer

The options of systemic treatment of patients with triple-negative BCBM are limited to conventional chemotherapy. Nevertheless, several drugs are investigated in this particularly challenging breast cancer subtype. Sacituzumab govitecan combines a humanized antibody against tumor-associated calcium signal transducer with the active metabolite of irinotecan. It is approved for the treatment of patients with metastatic triple-negative breast cancer [66]. The early results of a clinical trial NCT03995706 showed an intracranial activity of sacituzumab govitecan in patients with BCBM and recurrent glioblastoma [67].

Another investigated combination against triple-negative BCBM comprised iniparib and irinotecan [68]. Thirty-seven patients were enrolled. Thirty-four were eligible for evaluation. The median time to progression was 2 months. The median overall survival reached 8 months. Objective response to the treatment was observed in 12% of patients, whereas the clinical benefit rate was 27%. The toxicity of investigated regimen was acceptable.

An interesting concept of innovative drug delivery through the BBB was described by Zhang et al. [69]. The authors invented a terpolymer–lipid hybrid nanoparticle system for the treatment of integrin-overexpressing triple-negative BCBM. It was loaded with doxorubicin and mitomycin C. The system significantly reduced metastatic burden and increased host survival in in vitro and in vivo studies; however, it requires further development and clinical trials.

### 5.3. HER2-Positive Breast Cancer

HER2 tyrosine kinase inhibitors present intracranial activity in patients with HER2-positive BCBM. In the exploratory analysis of the HER2CLIMB clinical trial, the authors reported outcomes regarding intracranial efficacy and survival of patients with HER2-positive BCBM who received tucatinib or placebo, in combination with trastuzumab and capecitabine [70]. Among 291 patients enrolled in this trial, 108 had progressive BCBM. Patients with active BCBM, defined as progressive or untreated lesions, estimated that one year central nervous system progression-free survival was 35% in those who received tucatinib and 0% in patients who received placebo. This resulted in a 64% reduced risk of intracranial progression or death in the tucatinib arm compared to the placebo arm (hazard ratio 0.36, *p* < 0.0001) and translated into better overall survival in the subgroup who received tucatinib (20.7 months) versus placebo (11.6 months). Moreover, patients with HER2-positive BCBM who were treated with tucatinib showed more intracranial overall responses (47.3%) than those who took a placebo (20%, *p* = 0.03).

Lapatinib alone showed modest intracranial activity in a large phase II clinical trial that exclusively enrolled 242 patients with progressive HER2-positive BCBM who were previously treated with trastuzumab and radiotherapy [71]. Objective intracranial responses and a ≥20% volumetric reduction of brain lesions were noted in 6% and 21% of patients, respectively. The amendment allowed patients who progressed on lapatinib to receive lapatinib with capecitabine. Among 50 patients eligible for analysis, objective intracranial responses and ≥20% volumetric reductions of brain lesions were observed in 20% and 40%, respectively. Another phase I study with high dose lapatinib alternating with capecitabine also showed intracranial activity of such a combination [72].

Another HER2 tyrosine kinase inhibitor, neratinib, was investigated in combination with capecitabine in patients with progressive HER2-positive BCBM [73]. The patients were enrolled into two cohorts, namely, those who received previous treatment with lapatinib (cohort 3B) and lapatinib-naïve patients (cohort 3A). The primary endpoint of the study was the intracranial objective response rate calculated separately in each cohort. Objective response was defined as a reduction of 50% or more in the sum of target BCBM volumes without progression of nontarget lesions, new lesions, escalating steroids, progressive neurologic signs or symptoms, or extracranial progression. Cohort 3A enrolled 37 patients. Twelve patients entered cohort 3B. Intracranial objective response rates in cohort 3A and 3B were 49% and 33%, respectively. Median progression-free survival and overall survival reached 5.5 and 13.3 months in cohort 3A, respectively. Corresponding values for cohort 3B were 3.1 and 15.1 months, respectively. The most common grade 3 adverse event was diarrhea (29% in each cohort).

Trastuzumab emtansine presented a significant intracranial activity. In a single-arm phase IIIb clinical trial KAMILLA, response to the drug was observed in both previously irradiated and non-irradiated brain lesions [74]. However, the investigated drug was given to the patients with stable BCBM. Even better results were found in the recently published subgroup analysis from a phase III DESTINY-Breast03 trial. Patients with HER2-positive BCBM who received fam-trastuzumab deruxtecan-nxki (T-DXd) had significantly better intracranial response (intracranial response rate 63.8%) and 12 month progression-free survival (72%) in comparison with patients who were treated with trastuzumab emtansine (intracranial response rate of 33.3%, 12 month progression-free survival of 20.9%) [75]. Moreover, the group who received T-DXd had 75% lower relative risk of progression or death than patients from the trastuzumab emtansine group. Another salvage systemic treatment strategy could be the use of pertuzumab combined with high-dose trastuzumab [76]. Among 39 patients with progressive BCBM, such a treatment allowed for 11% of the intracranial overall response rate. Sixty-eight percent of patients experienced clinical benefit from the treatment, including two patients who had stable intracranial and extracranial disease for more than two years.

### 5.4. Hormone Receptor-Positive Breast Cancer

Few case series and case reports have shown the intracranial response of hormone receptor-positive BCBM [77,78,79]. However, no clinical trials or large retrospective studies have evaluated the efficacy of hormonal therapy as a salvage treatment for progressive BCBM. Thus, such a therapy should not be considered in this clinical situation. Cyclin-dependent kinase 4/6 inhibitors showed a moderate intracranial response in early phase clinical trials in patients with hormone receptor-positive BCBM [80,81].

## 6. Summary and Conclusions

**Figure 2:**
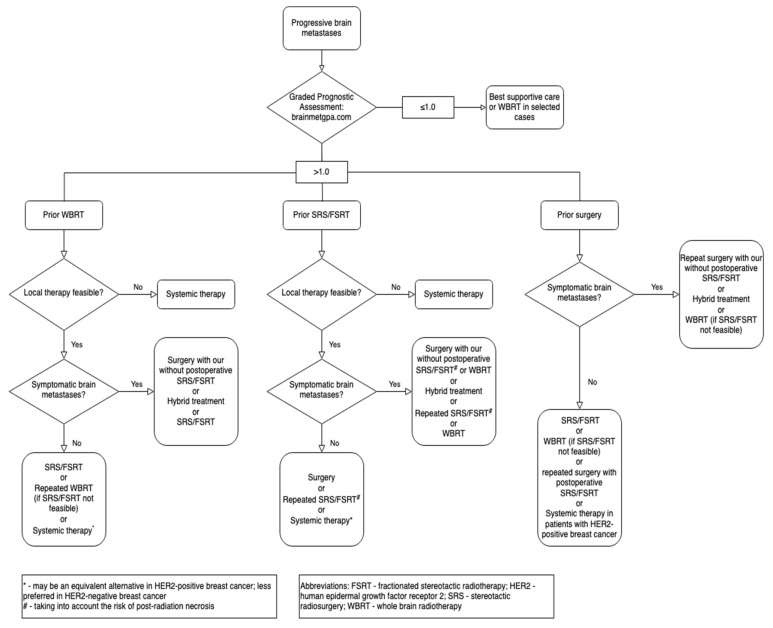
Salvage treatment methods for progressive brain metastases in breast cancer after prior local therapy.

Contemporary methods of local treatment for progressing BCBM provide good local efficacy and acceptable toxicity. The evolution of radiotherapy techniques allows for repeating focal treatment of multiple brain metastases. SRS or FSRT can be combined with surgery to increase intracranial control of the disease. SRS or FSRT can be repeated for previously irradiated volumes; however, this brings about a significant risk of PRN. Repeated WBRT could be offered to carefully selected patients with intracranial progression of numerous BCBM that are not amenable to SRS or FSRT. The benefit of WBRT is disputable. Systemic treatment may be an alternative option if local therapy is not feasible. The proposed methods of salvage treatment are summarized in Figure 2.

Nevertheless, the overall results of the salvage treatment of patients with progressive BCBM remain poor, mostly due to the progression outside treated lesions. There is a growing role of systemic treatment of progressing BCBM, including immunotherapy, targeted therapies, and special techniques of drug delivery. Patients with HER2-positive breast cancer have the widest scope of possible treatments that are effective, especially combinations of HER2 tyrosine kinase inhibitors with capecitabine and other agents. Systemic therapies may be combined with other available methods of local treatment to maximize survival. The selection of methods depends on performance status, disease burden, comorbidities, a biological subtype of breast cancer, and previously used therapies. The treatment decisions should be made at the multidisciplinary tumor board meetings that include at least neurosurgeon, radiation oncologist, and medical oncologist [82,83,84].

## Figures and Tables

**Table 1:** Fractionation regimens used in cranial radiosurgery and fractionated stereotactic radiotherapy.

Type of Radiotherapy	Dose Range (Gy)	Indication
SRS	20–24	max. tumor diameter ≤2.0 cm
	18	max. tumor diameter 2.1–3.0 cm
	15	max. tumor diameter 3.1–4.0 cm
FSRT	3 × 7–9 Gy	tumors larger than 2 cm
	5 × 5–7 Gy	

FSRT—fractionated stereotactic radiotherapy; SRS—stereotactic radiosurgery.

**Table 2:** Response Assessment in Neuro-Oncology criteria for brain metastases [17].

Response	Number of Met Criteria	Criteria
Complete response	All	No target lesions
		No non-target lesion
		No new lesions
		No deterioration of clinical status
		No steroids
Partial response	All	≥30% decrease in sum longest distance relative to baseline of target lesions
		At least stable non-target lesions
		No new lesions
		Stable or reduced steroids intake
		Stable or improved clinical status
Stable disease	All	<30% decrease relative to baseline but <20% increase in sum longest distance relative to nadir of target lesions
		At least stable non-target lesions
		No new lesions
		Stable or reduced steroid intake
		Stable or improved clinical status
Progressive disease	Any	≥20% increase in sum longest distance relative to nadir of target lesion
		unequivocal progression of existing enhancing or tumor-related non-enhancing (T2/FLAIR) non-target lesions
		New lesions (except patients who receive immunotherapy)
		Deteriorated clinical status

## Supplementary Materials

The following supporting information can be downloaded at: https://www.mdpi.com/article/10.3390/cancers14041096/s1. Table S1: Methods of salvage treatment in breast cancer brain metastases—analysis of available data and the quality of evidence.
