# Supplementary material for: Salvage Treatment for Progressive Brain Metastases in Breast Cancer
Source: Cancers (Basel). 2022 Feb 21;14(4):1096. doi: 10.3390/cancers14041096 (PMC8870695; doi:10.3390/cancers14041096)
Supplement: Supplementary file 1 [file cancers-14-01096-s001.zip › cancers-1519134-supplementary.pdf]

Supplementary Materials

Salvage Treatment for Progressive Brain Metastases in Breast Cancer

Mateusz Jacek Spałek [1,*] and Tomasz Mandat [2]

Table S1. Methods of salvage treatment in breast cancer brain metastases – analysis of available data and the quality of evidence.

Publication	Type of study	Group who received salvage treatment for brain metastases	Previous therapy for breast cancer brain metastases	Intervention	Oxford Centre for Evidence-Based Medicine Level of Evidence (1 - high; 2 - moderate; 3-5 - low)
Combs SE, Schulz-Ertner D, Thilmann C, Edler L, Debus J. Treatment of cerebral metastases from breast cancer with stereotactic radiosurgery. Strahlenther Onkol. 2004 Sep;180(9):590-6. doi: 10.1007/s00066-004-1299-x. PMID: 15378190.	Retrospective cohort study	Breast cancer only (*n* = 39)	Whole brain radiotherapy	Stereotactic radiosurgery	2b
Akyurek S, Chang EL, Mahajan A, Hassenbusch SJ, Allen PK, Mathews LA, Shiu AS, Maor MH, Woo SY. Stereotactic radiosurgical treatment of cerebral metastases arising from breast cancer. Am J Clin Oncol. 2007 Jun;30(3):310-4. doi: 10.1097/01.coc.0000258365.50975.f6. PMID: 17551311.	Retrospective cohort study	Breast cancer only (*n* = 15)	Whole brain radiotherapy	Stereotactic radiosurgery	2b
Kased N, Binder DK, McDermott MW, Nakamura JL, Huang K, Berger MS, Wara WM, Sneed PK. Gamma Knife radiosurgery for brain metastases from primary breast cancer. Int J	Retrospective cohort study	Breast cancer only (*n* = 103)	Whole brain radiotherapy (*n* = 81); whole brain radiotherapy	Stereotactic radiosurgery	2b

Radiat Oncol Biol Phys. 2009 Nov 15;75(4):1132-40. doi: 10.1016/j.ijrobp.2008.12.031. Epub 2009 Apr 3. PMID: 19345514.			and surgery (*n* = 18); surgery alone (*n* = 4)		
Kelly PJ, Lin NU, Claus EB, Quant EC, Weiss SE, Alexander BM. Salvage stereotactic radiosurgery for breast cancer brain metastases: outcomes and prognostic factors. Cancer. 2012 Apr 15;118(8):2014-20. doi: 10.1002/cncr.26343. Epub 2011 Sep 14. PMID: 21918959.	Retrospective cohort study	Breast cancer only (*n* = 79)	Whole brain radiotherapy (*n* = 63); whole brain radiotherapy and surgery (*n* = 13); surgery alone (*n* = 1); total meningeal irradiation (*n* = 1); systemic treatment (*n* = 1)	Stereotactic radiosurgery	2b
Huang Z, Sun B, Shen G, Cha L, Meng X, Wang J, Zhou Z, Wu S. Brain metastasis reirradiation in patients with advanced breast cancer. J Radiat Res. 2017 Jan;58(1):142-148. doi: 10.1093/jrr/rrw087. Epub 2016 Oct 5. PMID: 27707842; PMCID: PMC5321192.	Retrospective cohort study	Breast cancer only (*n* = 56)	Whole brain radiotherapy (*n* = 39); stereotactic radiosurgery (*n* = 17)	Stereotactic radiosurgery after whole brain radiotherapy; whole brain radiotherapy after stereotactic surgery	2b
Lai, S. & Huang, C. & Wang, C. & Chen, Yitian & Lan, K. & Cheng, A. & Kuo, S.. (2013). Brain Re-irradiation for Breast Cancer Patients With Brain Metastasis After Whole Brain Radiation Therapy: Effect of Epidermal Growth Factor Receptor-2 Status on Clinical Outcome. International Journal of Radiation Oncology*Biology*Physics. 87. S243. 10.1016/j.ijrobp.2013.06.632.	Retrospective cohort study	Breast cancer only (*n* = 40)	Whole brain radiotherapy	Stereotactic radiosurgery; fractionated stereotactic radiotherapy; repeated whole brain radiotherapy	2b
Kano H, Kondziolka D, Zorro O, Lobato-Polo J, Flickinger JC, Lunsford LD. The results of resection after stereotactic radiosurgery for brain	Retrospective cohort study (subgroup)	Various cancers including *n* = 9 patients with breast cancer	Stereotactic radiosurgery	Resection	4

metastases. J Neurosurg. 2009 Oct;111(4):825-31. doi: 10.3171/2009.4.JNS09246. PMID: 19425892.					
Truong MT, St Clair EG, Donahue BR, Rush SC, Miller DC, Formenti SC, Knopp EA, Han K, Golfinos JG. Results of surgical resection for progression of brain metastases previously treated by gamma knife radiosurgery. Neurosurgery. 2006 Jul;59(1):86-97; discussion 86-97. doi: 10.1227/01.NEU.0000219858.80351.38. PMID: 16823304.	Retrospective cohort study (subgroup)	Various cancers including *n* = 4 patients with breast cancer	Stereotactic radiosurgery	Resection	4
Mitsuya K, Nakasu Y, Hayashi N, Deguchi S, Oishi T, Sugino T, Yasui K, Ogawa H, Onoe T, Asakura H, Harada H. Retrospective analysis of salvage surgery for local progression of brain metastasis previously treated with stereotactic irradiation: diagnostic contribution, functional outcome, and prognostic factors. BMC Cancer. 2020 Apr 17;20(1):331. doi: 10.1186/s12885-020-06800-w. PMID: 32303195; PMCID: PMC7165413.	Retrospective cohort study (subgroup)	Various cancers including *n* = 9 patients with breast cancer	Stereotactic radiosurgery	Resection with or withour postoperative radiotherapy	4
McKay WH, McTyre ER, Okoukoni C, Alphonse-Sullivan NK, Ruiz J, Munley MT, Qasem S, Lo HW, Xing F, Laxton AW, Tatter SB, Watabe K, Chan MD. Repeat stereotactic radiosurgery as salvage therapy for locally recurrent brain metastases previously treated with radiosurgery. J Neurosurg. 2017 Jul;127(1):148-156. doi: 10.3171/2016.5.JNS153051. Epub 2016 Aug 5. PMID: 27494815.	Retrospective cohort study (subgroup)	Various cancers including *n* = 9 patients with breast cancer	Stereotactic radiosurgery	Stereotactic radiosurgery	4

Rana N, Pendyala P, Cleary RK, Luo G, Zhao Z, Chambless LB, Cmelak AJ, Attia A, Stavas MJ. Long-term Outcomes after Salvage Stereotactic Radiosurgery (SRS) following In-Field Failure of Initial SRS for Brain Metastases. Front Oncol. 2017 Nov 23;7:279. doi: 10.3389/fonc.2017.00279. PMID: 29218301; PMCID: PMC5703829.	Retrospective cohort study (subgroup)	Various cancers including *n* = 5 patients with breast cancer	Stereotactic radiosurgery	Stereotactic radiosurgery	4
Lucia F, Touati R, Crainic N, Dissaux G, Pradier O, Bourbonne V, Schick U. Efficacy and Safety of a Second Course of Stereotactic Radiation Therapy for Locally Recurrent Brain Metastases: A Systematic Review. Cancers (Basel). 2021 Sep 30;13(19):4929. doi: 10.3390/cancers13194929. PMID: 34638412; PMCID: PMC8508410.	Systematic review of cohort studies	Various cancers including *n* = 61 patients with breast cancer	Stereotactic radiosurgery	Stereotactic radiosurgery	2a
Guo, S.; Reddy, C.A.; Chao, S.T.; Suh, J.H. Repeat Whole Brain Irradiation for Patients with Brain Metastases. International Journal of Radiation Oncology, Biology, Physics 2011, 81, S645, doi:10.1016/j.ijrobp.2011.06.1906.	Retrospective cohort study (subgroup)	Various cancers including *n* = 8 patients with breast cancer	Whole brain radiotherapy	Whole brain radiotherapy	4
Boogerd W, Dalesio O, Bais EM, van der Sande JJ. Response of brain metastases from breast cancer to systemic chemotherapy. Cancer. 1992 Feb 15;69(4):972-80. doi: 10.1002/1097-0142(19920215)69:4<972::aid-cncr2820690423>3.0.co;2-p. PMID: 1735089.	Retrospective cohort study (subgroup)	Breast cancer (*n* = 9)	Surgery and/or radiotherapy	Cyclophosphamide, methotrexate, and 5-fluorouracil or cyclophosphamide, doxorubicin, and 5-fluorouracil	4
Christodoulou C, Bafaloukos D, Linardou H, Aravantinos G, Bamias A, Carina M, Klouvas G, Skarlos D; Hellenic Cooperative Oncology	Prospective nonrandomized phase 2 clinical trial (subgroup)	Various cancers including *n* = 15 patients with breast cancer	Any form of local treatment	Temozolomide	2b

Group. Temozolomide (TMZ) combined with cisplatin (CDDP) in patients with brain metastases from solid tumors: a Hellenic Cooperative Oncology Group (HeCOG) Phase II study. J Neurooncol. 2005 Jan;71(1):61-5. doi: 10.1007/s11060-004-9176-0. PMID: 15719277.					
Rivera E, Meyers C, Groves M, Valero V, Francis D, Arun B, Broglio K, Yin G, Hortobagyi GN, Buchholz T. Phase I study of capecitabine in combination with temozolomide in the treatment of patients with brain metastases from breast carcinoma. Cancer. 2006 Sep 15;107(6):1348-54. doi: 10.1002/cncr.22127. PMID: 16909414.	Prospective non-randomized phase 2 clinical trial (subgroup)	Breast cancer (*n* = 10)	Any form of local treatment	Temozolomide and capecitabine	2b
Berghoff AS, Sax C, Klein M, Furtner J, Dieckmann K, Gatterbauer B, Widhalm G, Rudas M, Zielinski CC, Bartsch R, Preusser M. Alleviation of brain edema and restoration of functional independence by bevacizumab in brain-metastatic breast cancer: a case report. Breast Care (Basel). 2014 May;9(2):134-6. doi: 10.1159/000360930. PMID: 24944558; PMCID: PMC4038309.	Case report	Breast cancer	Surgery, stereotactic radiosurgery and whole brain radiotherapy	Bevacizumab	4
Berghoff AS, Breckwoldt MO, Riedemann L, Karimian-Jazi K, Loew S, Schlieter F, Furtner J, Cinci M, Thomas M, Strowitzki MJ, Marmé F, Michel LL, Schmidt T, Jäger D, Bendszus M, Preusser M, Wick W, Winkler F. Bevacizumab-based treatment as salvage therapy in patients with recurrent symptomatic brain metastases. Neurooncol Adv. 2020 Mar 16;2(1):vdaa038. doi:	Retrospective cohort study	Breast cancer (*n* = 22)	Any form of local treatment	Bevacizumab	2b

10.1093/noajnl/vdaa038. PMID: 32642693; PMCID: PMC7212911.					
Brenner, A.J.; Pandey, R.; Chiou, J.; Floyd, J.; Garcia, M.; Surapaneni, P.; Kaklamani, V.; Lathrop, K.; Crownover, R.; Caron, J.L.; et al. 373MO Delivery and Activity of SN-38 by Sacituzumab Govitecan in CNS Tumours. Annals of Oncology 2020, 31, S401, doi:10.1016/j.annonc.2020.08.482.	Prospective single arm window of opportunity trial	Breast cancer (*n* = 7)	Any form of local treatment	Sacituzumab govitecan	2b
Anders C, Deal AM, Abramson V, Liu MC, Storniolo AM, Carpenter JT, Puhalla S, Nanda R, Melhem-Bertrandt A, Lin NU, Kelly Marcom P, Van Poznak C, Stearns V, Melisko M, Smith JK, Karginova O, Parker J, Berg J, Winer EP, Peterman A, Prat A, Perou CM, Wolff AC, Carey LA. TBCRC 018: phase II study of iniparib in combination with irinotecan to treat progressive triple negative breast cancer brain metastases. Breast Cancer Res Treat. 2014 Aug;146(3):557-66. doi: 10.1007/s10549-014-3039-y. Epub 2014 Jul 8. PMID: 25001612; PMCID: PMC4112043.	Prospective non-randomized phase 2 clinical trial	Breast cancer (*n* = 37)	Any form of local treatment	Iniparib and irinotecan	2b
Lin NU, Borges V, Anders C, Murthy RK, Paplomata E, Hamilton E, Hurvitz S, Loi S, Okines A, Abramson V, Bedard PL, Oliveira M, Mueller V, Zelnak A, DiGiovanna MP, Bachelot T, Chien AJ, O'Regan R, Wardley A, Conlin A, Cameron D, Carey L, Curigliano G, Gelmon K, Loibl S, Mayor J, McGoldrick S, An X, Winer EP. Intracranial Efficacy and Survival With Tucatinib Plus Trastuzumab and Capecitabine for	Prospective randomized controlled trial	Breast cancer (*n* = 108)	Any form of treatment	Tucatinib or placebo, in combination with trastuzumab and capecitabine	1b

Previously Treated HER2-Positive Breast Cancer With Brain Metastases in the HER2CLIMB Trial. J Clin Oncol. 2020 Aug 10;38(23):2610-2619. doi: 10.1200/JCO.20.00775. Epub 2020 May 29. PMID: 32468955; PMCID: PMC7403000.					
Lin NU, Diéras V, Paul D, Lossignol D, Christodoulou C, Stemmler HJ, Roché H, Liu MC, Greil R, Ciruelos E, Loibl S, Gori S, Wardley A, Yardley D, Brufsky A, Blum JL, Rubin SD, Dharan B, Steplewski K, Zembryki D, Oliva C, Roychowdhury D, Paoletti P, Winer EP. Multicenter phase II study of lapatinib in patients with brain metastases from HER2-positive breast cancer. Clin Cancer Res. 2009 Feb 15;15(4):1452-9. doi: 10.1158/1078-0432.CCR-08-1080. PMID: 19228746.	Prospective non-randomized phase 2 clinical trial	Breast cancer (*n* = 242)	Radiotherapy and trastuzumab	Lapatinib	2b
Morikawa A, de Stanchina E, Pentsova E, Kemeny MM, Li BT, Tang K, Patil S, Fleisher M, Van Poznak C, Norton L, Seidman AD. Phase I Study of Intermittent High-Dose Lapatinib Alternating with Capecitabine for HER2-Positive Breast Cancer Patients with Central Nervous System Metastases. Clin Cancer Res. 2019 Jul 1;25(13):3784-3792. doi: 10.1158/1078-0432.CCR-18-3502. Epub 2019 Apr 15. PMID: 30988080; PMCID: PMC6773251.	Prospective phase 1 clinical trial	Breast cancer	Any form of treatment	High dose lapatinib alternating with capecitabine	2b
Freedman RA, Gelman RS, Anders CK, Melisko ME, Parsons HA, Cropp AM, Silvestri K, Cotter CM, Componeschi KP, Marte JM, Connolly RM, Moy B, Van Poznak CH, Blackwell KL, Puhalla SL, Jankowitz	Prospective non-randomized phase 2 clinical trial	Breast cancer (*n* = 49)	Any form of treatment	Neratinib	2b

RC, Smith KL, Ibrahim N, Moynihan TJ, O'Sullivan CC, Nangia J, Nira-vath P, Tung N, Pohlmann PR, Burns R, Rimawi MF, Krop IE, Wolff AC, Winer EP, Lin NU; Translational Breast Cancer Research Consortium. TBCRC 022: A Phase II Trial of Neratinib and Capecitabine for Pa-tients With Human Epidermal Growth Factor Receptor 2-Positive Breast Cancer and Brain Metastases. J Clin Oncol. 2019 May 1;37(13):1081-1089. doi: 10.1200/JCO.18.01511. Epub 2019 Mar 12. PMID: 30860945; PMCID: PMC6494354.					
Lin NU, Pegram M, Sahebjam S, Ib-rahim N, Fung A, Cheng A, Nicholas A, Kirschbrown W, Kumthekar P. Pertuzumab Plus High-Dose Trastuzumab in Patients With Pro-gressive Brain Metastases and HER2-Positive Metastatic Breast Cancer: Primary Analysis of a Phase II Study. J Clin Oncol. 2021 Aug 20;39(24):2667-2675. doi: 10.1200/JCO.20.02822. Epub 2021 May 4. PMID: 33945296; PMCID: PMC8376355.	Prospective non-randomized phase 2 clinical trial	Breast cancer (*n* = 39)	Radiotherapy	Pertuzumab and high-dose trastuzumab	2b
Stewart DJ, Dahrouge S. Response of brain metastases from breast cancer to megestrol acetate: a case report. J Neurooncol. 1995;24(3):299-301. doi: 10.1007/BF01052847. PMID: 7595761.	Case report	Breast cancer	Resection, ra-diotherapy	Megestrol acetate	4
Salvati M, Cervoni L, Innocenzi G, Bardella L. Prolonged stabilization of multiple and single brain metastases from breast cancer with tamoxifen. Report of three cases. Tumori. 1993	Case series	Breast cancer (*n* = 3)	Unknown	Tamoxifen	4

Oct 31;79(5):359-62. PMID: 8116083.					
Tolaney SM, Sahebjam S, Le Rhun E, Bachelot T, Kabos P, Awada A, Yardley D, Chan A, Conte P, Diéras V, Lin NU, Bear M, Chapman SC, Yang Z, Chen Y, Anders CK. A Phase II Study of Abemaciclib in Patients with Brain Metastases Secondary to Hormone Receptor-Positive Breast Cancer. Clin Cancer Res. 2020 Oct 15;26(20):5310-5319. doi: 10.1158/1078-0432.CCR-20-1764. Epub 2020 Jul 21. Erratum in: Clin Cancer Res. 2021 Mar 1;27(5):1582. PMID: 32694159.	Prospective non-randomized phase 2 clinical trial	Breast cancer (*n* = 104; majority of them received previous treatment for brain metatases)	Any form of treatment	Abemaciclib	2b
